# A Critical Assessment of Friedenwald's Technique for Estimating the Coefficient of Rigidity of the Cornea

**DOI:** 10.1155/2022/6775064

**Published:** 2022-10-04

**Authors:** Larysa Tutchenko, Sudi Patel, Mykhailo Skovron, Olha Horak, Oleksiy Voytsekhivskyy

**Affiliations:** ^1^Kyiv City Clinical Ophthalmological Hospital “Eye Microsurgical Center”, Kyiv, Ukraine; ^2^Specialty Eye Hospital Svjetlost, Heinzelova 39, Zagreb 10000, Croatia

## Abstract

**Purpose:**

To determine if Friedenwald's technique for estimating the coefficient of corneal rigidity (Ko, units mmHg/*μ*L), could differentiate between the cornea in keratoconus, normal eyes, and after crosslinking (CXL).

**Methods:**

Two operators (1 and 2) independently measured Ko in three groups (keratoconus, normal, and post-CXL corneas), and repeated the procedure in some where their care remained unchanged and others after routine CXL (>28 days postop, epi-off treatment, 3.0 mW/cm^2^, 30 min). The data were subsequently used to quantify interoperator error, test-retest/intersessional reliability for estimation of Ko, the significance of intergroup differences, and the effect of CXL on Ko.

**Results:**

The major findings were: (i) Ko values were not normally distributed; (ii) mean (±sd, 95% CI) interoperator error was -0.002 (±0.019, −0.006 to 0.003, *n* = 95) and the limit of agreement between the operators was ±0.039; (iii) RMS differences in the intersessional estimation of Ko values were 0.011 (operator 1) and 0.012 (operator 2); (iv) intergroup differences in Ko were not significant (*p* > 0.05); (v) intersessional change in Ko (*y*) was linearly related to Ko estimated (*x*) at 1^st^ session (for operator 2 *y* = 1.187*x*−0.021, *r* = 0.755, *n* = 16, *p* < 0.01); and (vi) change in Ko (*y*_1_) after CXL was linearly related to Ko (*x*_1_) at preop (for operator 2 *y*_1_ = 0.880*x*_1_−0.016, *r* = 0.935, *n* = 20, *p* < 0.01).

**Conclusion:**

Friedenwald's technique for estimating the Ko is prone to substantial interoperator error and intersessional differences. According to the technique, the change in Ko following CXL is on par with the expected intersessional change observed in controls.

## 1. Introduction

The Schiøtz tonometer was introduced in 1905 and quickly gained popularity for the measurement of intraocular pressure [1]. Friedenwald modified the procedure underpinning the use of the Schiøtz tonometer to estimate the rigidity of the cornea [2, 3]. He formulated a nomogram for determining the coefficient of corneal rigidity (Ko, units mmHg/*μ*L), and printed versions of the nomogram were included with new models of the Schiøtz tonometer. Friedenwald's original nomogram was a graph featuring a quadrant marked with Ko values and four descending curves marked off with scale readings. The geometric centre of the quadrant is coincident with the origin of the graph, and each descending curve features scale readings for each of the four tonometer weights (5.5 g, 7.5 g, 10.0 g, and 15.0 g). Two Schiøtz tonometer scale readings (for example, obtained using the 5.5 g and 7.5 g weights) are marked on the nomogram. A straight line is drawn connecting the two marks. A second line is drawn parallel to the first and passes through the origin of the graph. The figure closest to the point where the second line traverses the quadrant is the Ko value for the case in hand. The slope of this line is the determinate of Ko. The method is akin to linear regression using two values. Some investigators used three weights (e.g., 5 g, 7.5 g, and 10.0 g) to improve the precision of Ko estimation [4–7]. The basic premise of Friedenwald's technique, termed differential tonometry, has been critically reviewed in various texts [8–10]. Duke-Elder commented on the validity of the nomogram, noting that a half-unit error when reading a Schiøtz tonometer scale results in a substantial error in Ko estimation, especially when the true rigidity of the cornea is unusually high [9]. Nevertheless, the technique proved popular and has been used to estimate Ko in a diverse range of conditions, including keratoconus, myopia, precorneal and postcorneal refractive procedures, disease-free and health conditions such as osteogenesis imperfecta [2, 4–6, 11–15].

Clinical instruments are manufactured and calibrated to exacting standards. However, this does not guarantee an instrument is viable and trustworthy. Hence, it is customary to evaluate the clinical efficacy of an instrument by checking, for example, its validity, repeatability, sensitivity, and specificity. It would be helpful to understand the interoperator error, or dissimilarities, in the estimation of Ko using Friedenwald's technique. In addition, it would also be useful to have an indication of the test-retest/intersessional repeatability in the estimation of Ko if the technique could differentiate between normal and keratoconus eyes and detect any differences in Ko following surgical intervention designed to improve the rigidity in keratoconus eyes.

A literature search failed to identify any published reports on the interoperator error and repeatability in the estimation of Ko or whether Ko changes after surgical intervention in keratoconus eyes. Corneal crosslinking (CXL) is an effective treatment for strengthening the cornea in keratoconus, and this has been covered in several review papers [16–21]. Bench tests on human and other mammalian corneas have consistently shown that CXL renders the cornea more resistant to deforming forces [22–27]. Thus, it would be useful to determine if Friedenwald's technique can detect any alterations in Ko following CXL treatment for keratoconus.

The aim of this study was to determine

- the repeatability of Ko estimation, by considering the interoperator error and test-retest/intersessional consistency in the estimation of Ko,
- if Friedenwald's technique could detect alterations in Ko following CXL treatment of keratoconus.

## 2. Materials and Methods

### 2.1. Calculation of Ko from Details on Friedenwald's Nomogram

A programme for the calculation of Ko was constructed using the details and dimensions of Friedenwald's printed nomogram. This calculated the slope of the regression line that would have been hand drawn on the paper nomogram, connecting the scale readings for each of the permutations using either 2, 3, or all 4 Schiøtz tonometer (corresponding to scale readings obtained using the 5.5 g, 7.5 g, 10.0 g, and 15.0 g) weights. The corresponding Ko value is then identified as it is conjugate with the gradient of the slope.

### 2.2. Estimation of Ko Using Schiøtz Tonometer

A brand new, previously unused, factory-calibrated Schiøtz tonometer (Gulden Ophthalmics, Elkins Park, PA) was used in all cases. Ko was measured with the patient in the supine position following topical anaesthesia with proparacaine hydrochloride 0.5% drops. Before any assessment, the tonometer was cleaned and sterilised as follows: soaked for 5 min in 3% hydrogen peroxide, rinsed thoroughly with sterile saline, soaked for 5 min in 70% ethanol, in turn, rinsed and dipped into sterile saline, and dried. When ready, the Schiøtz tonometer was placed at the centre of the cornea, and the scale reading was recorded immediately (5.5 g plunger weight). With the tonometer remaining steady on the cornea, the scale readings were recorded after another operator sequentially increased the plunger weights to 7.5 g, 10.0 g, and 15.0 g. During this time, the operator held the subject's eyelids open with the thumb and index finger of one hand and kept the tonometer on the subject's eye with the other. The tonometer was removed, the subject was asked to relax, and remain in the supine position for 5 minutes, and the procedure was performed on the left eye. The total time for taking a series of readings on one eye was about 20 seconds. The approximate scale reading was recorded when there was some oscillation of the pointer. These data were then used to estimate Ko using the custom programme.

### 2.3. Study Design and Criteria for Allocation of Subjects

The investigation was a prospective, consecutive, randomized, masked, observational study approved by the prevailing Ethics Board and followed the tenets of the Declaration of Helsinki. All subjects signed consent forms after the aims and procedures of the investigation were fully explained.

Data were harvested from three groups: [A] previously diagnosed keratoconus and later elected for crosslinking during the study; [B] patients that had undergone crosslinking (post-CXL); and [C] normal controls.

The diagnosis of keratoconus (group A) was based on a combination of signs revealed during refraction (such as presence of scissor retinoscopic reflex, manifest refractive astigmatism increasing by ≥ 1D, and deterioration of best corrected visual acuity over the previous year), biomicroscopy (Vogt striae, Munson's sign, corneal iron lines, and Fleischer ring), tomography, topography (posterior surface elevation >15 *μ*m, corneal steepening >48D, skewed radial axis >22°, inferior-superior asymmetry >1.4D), and pachymetry (corneal thinning in the paracentral and/or inferior regions, central corneal thickness <480 *μ*m, difference in corneal thickness between the corneal apex and the thinnest region>10 *μ*m). All patients fulfilled the criteria for CXL. They were progressive keratoconus because either the Kmax or manifest refractive astigmatism had increased by ≥ 1D, and best corrected visual acuity had deteriorated over the previous year. The exclusion criteria were corneas thinner than 380 *μ*m, history of previous corneal surgical intervention, corneal scarring, severe infection, or other corneal diseases. Where appropriate, patients were asked to discontinue wearing any rigid contact lenses for at least three weeks (one week for soft lenses) before the acquisition of any study data. None were classified as mild subclinical cases of keratoconus.

The post-CXL cases (group B) consisted of patients attending for routine follow-up checks. Measurements were taken from each subject on a consecutive, case-by-case, basis. Patients were excluded where there was a history of corneal surgical interventions other than CXL, ocular surface infection, or other corneal diseases.

The normal control group (group C) were healthy age/gender matched volunteers with no signs of keratoconus, no history of contact lens wear, no ocular conditions, or history of systemic health conditions known to affect the cornea (such as corneal dystrophy or scar, keratitis, allergic conjunctivitis, rheumatism, diabetes).

All subjects underwent a full ophthalmological examination that included noncontact pachymetry, tomography, and pneumotonometry before estimation of Ko.

The investigation was in two parts, as follows:

PartI, interoperator error and test-retest/intersessional consistency in the estimation of Ko.

- Two operators (labelled 1 and 2) underwent a period of training and were directed to use the Schiøtz tonometer to obtain scale readings from three separate groups (A, B, and C). Measurements were taken from the right eye, then the left eye after a break of approximately 5 minutes. Where applicable, measurements were taken from the treated eye in post-CXL cases and the untreated eye in keratoconus subjects. The interval between measurements taken by the two operators was 15 minutes.
- The operators were instructed to repeat measurements on the patients they had checked previously when the patients returned for routine appointments. These were patients for whom the care regime remained unchanged during the intervening period. The operators were kept unaware of their previous findings during the repeat session. 

Part II, estimation of Ko before and after CXL treatment for keratoconus.

The operators were instructed to take measurements from the keratoconus patients they had checked previously, after the patients had received crosslinking without complications. The operators were kept unaware of their previous findings during the repeat session.

### 2.4. Description of Crosslinking Procedure and Postoperative Treatment

Corneal crosslinking (epi-off, 3.0 mW/cm^2^, 30 min) was performed by one surgeon (LT). Topical anaesthesia was made with proparacaine hydrochloride 0.5% drops. The corneal epithelium was debrided over the central 8 mm zone after soaking with 20% alcohol for 30 sec. This was followed by the instillation of riboflavin 0.1% with 20% dextran onto the cornea every 2 min for a total of 30 minutes. If the corneal thickness (measured by using Axis II PR Ultrasound A mode and Pachymeter, Quantel Medical) at this point was 400 *μ*m or more, then it was exposed to UVA radiation at a wavelength of near 370 nm and an irradiance of 3.0 mW/cm^2^ with simultaneously continued instillation of riboflavin 0.1% with 20% dextran on the cornea every 2 minutes for a total of 30 minutes. If the corneal thickness before irradiation was thinner than 400 *μ*m, the cornea was hydrated with hypotonic riboflavin until the pachymetry measured a minimum of 400 *μ*m. A soft bandage contact lens was placed over the cornea at the end of the procedure and remained on the patient's eye until re-epithelialization had been completed. Postoperative treatment included drops of levofloxacin, dexamethasone, and dexpanthenol gel 5 times a day each with a gradual taper off, and a preservative-free combination of trehalose and hyaluronic acid 3 times a day.

### 2.5. Statistical Analysis

The data were stored on an Excel spreadsheet (Microsoft, Redmond, WA) and analysed as follows:

  Part 1.
  To determine the significance of any variations in Ko values obtained by the two operators using the method of Bland and Altman [28], and the significance of any apparent differences in Ko values between sessions (Wilcoxon signed-rank test and paired *t*-test). If significant variations were found, then the analysis would be extended to determine if the Ko values acquired by each operator could distinguish between keratoconus, normal, and post-CXL cases (Kruskal–Wallis test and 1-way ANOVA).
  Part 2.
  To determine the significance of any differences in Ko values before and after routine CXL (Wilcoxon signed-rank test and paired *t*-test), and if any change was associated with the estimated Ko before CXL (Pearson correlation coefficient [*r*]), and if any change was correlated with the time elapsed since CXL was treatment was completed (Pearson correlation coefficient [*r*]).
  The three groups (keratoconus, controls, and post-CXL cases) were compared for differences in age, IOP, and pachymetry.
  Nonparametric tests were applied when data were not normally distributed (Kolmogorov–Smirnov test of normality). Changes and differences were considered statistically significant when *p* < 0.05.

## 3. Results

**Figure 1:**
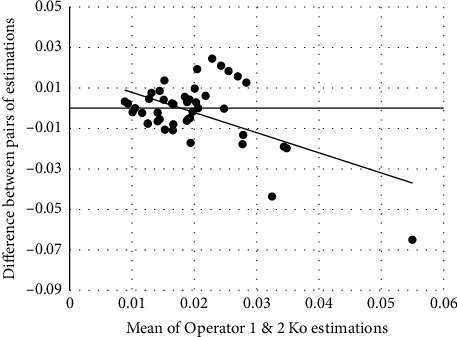
Bland and Altman plot for Ko. The solid line represents the least squares regression line.

**Figure 2:**
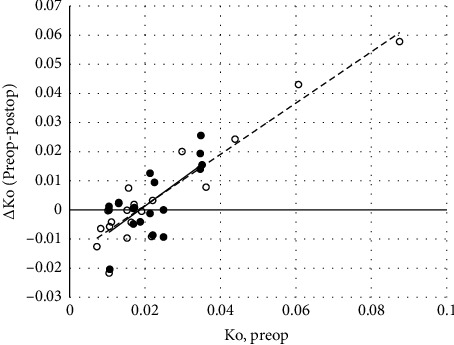
Change in Ko and preop Ko. The filled and empty circles are, respectively, the results according to measurements obtained by operators 1 and 2. The solid and dashed lines are the corresponding least squares regression lines.

All permutations for the calculation of Ko using the four weights were investigated during the preliminary analysis of the data. There were no useful statistical advantages gained by using the data obtained from any of the combinations of weights over and above the data obtained using 5.5, 7.5, and 10 g weights. The Ko values in Tables 1–3 and Figures 1 and 2 were derived after omitting scale readings obtained using the 15 g weight. There were no significant differences in IOP between the three groups of subjects examined by either operator 1 or 2 (1-way ANOVA, *p* > 0.05). All Ko values are expressed in units of mmHg/*μ*L.

### 3.1. Interoperator Error

The operators took measurements from a total of 33 keratoconus subjects (51 eyes of 10 females and 23 males), 29 post-CXL subjects (40 eyes of 9 females and 20 males), and 10 normal controls (20 eyes of 5 females and 5 males). The mean (±sd, 95% confidence interval) period between CXL treatment and estimation of Ko in the post-CXL patients was 12.3 months (±17.9, 6.1–18.4). The mean (±sd, 95% confidence interval) age (years) of the subjects in the 3 groups (keratoconus, post-CXL and normal controls) were 25.7 (±7.5, 15–47), 27.1 (±6.1, 17–43), and 31.7 (±11.5, 20–57). Pachymetry values (*μ*m) were 419.4 (±44.2, 406.8–432.1), 342.9 (±58.9, 323.8–361.9), and 549.8(±36.3, 524.6–575.0). Apparent differences were not significant for age (1-way ANOVA, *F* = 2.05, *p* > 0.05), but were for pachymetry (1-way ANOVA, *F* = 63.23, *p* < 0.01).  Table 1 shows the frequency distribution of Ko values. Clearly, values ≥ 0.11 were outside of the mainstream. The Ko values were not normally distributed (*p* < 0.01). The same was encountered after excluding Ko values ≥ 0.11 except for the results according to measurements obtained by operator 1 for the normal control group (*p* = 0.13).

Figure 1 is a Bland and Altman plot showing the interoperator errors or differences in Ko estimates after excluding pairs of values where at least one of the pair of Ko values was ≥0.11. The mean difference (±sd, 95% confidence interval) between individual pairs of measurements was −0.002 (±0.019, −0.006 to 0.003) and the limit of agreement (±1.96sd) between the two operators was ±0.039. There was a significant correlation between the difference in each pair of estimations (*y*) and the mean of the pair of estimations obtained by the operators (*x*). The association between *x* and *y* is described by

(1) $$\begin{array}{c} Y=0.018-0.996x\left(r=-0.532,n=95,p<0.01\right). \end{array}$$

### 3.2. Interoperator and Intergroup Differences in the Estimation of Ko

Table 2 shows the main details of the Ko estimates obtained by the operators after excluding all cases where at least one of a pair of Ko estimates was ≥0.11. Neither interoperator differences (Wilcoxon signed-rank test. Keratoconus, *z* = −0.31, *p*=0.76. Post-CXL, *z* = −0.87, *p*=0.38. Controls, *z* = −1.25, *p*=0.21) nor intergroup differences (Kruskal–Wallis test. Operator 1, *H* = 1.24, *p*=0.54. Operator 2, *H* = 1.80, *p*=0.41) in the estimation of Ko were significant.

### 3.3. Intersessional Error

Table 3 shows the main details of the repeat estimations of Ko values. Operator 1 repeated measurements on 27 eyes (13 keratoconus and 14 post-CXL eyes of 3 females and 19 males, age range 20–33 years) and operator 2 repeated measurements on 16 eyes (13 keratoconus and 3 post-CXL eyes of 2 females and 13 males, age range 20–31 years). Apparent intersessional differences in Ko values were not significant (Wilcoxon signed-rank test. Operator 1, *z* = −1.49, *p*=0.14. Operator 2, *z* = −0.47, *p*=0.64). The RMS difference (±sd) in Ko values between sessions 1 and 2 were 0.011 (±0.013, *n* = 27) for operator 1 and 0.012 (±0.013, *n* = 16) for operator 2.

Linear regression revealed a significant association between the difference in Ko values between sessions 1 and 2 (*y*) and the Ko revealed during session 1 (*x*) for operator 2. The association between *x* and *y* is described

(2) $$\begin{array}{c} Y=1.187x-0.021\left(r=0.755,n=16,p<0.01\right). \end{array}$$

A similar significant correlation was not revealed for the results obtained by operator 1.

#### 3.3.1. Change in Ko after CXL

Pre- and post-CXL Ko values were obtained from 18 eyes (5 females and 11 males, age range 20–37 years) by operator 1 and from 20 eyes (5 females and 12 males, age range 20–33 years) by operator 2. Apparent changes in the Ko values were not significant (Wilcoxon signed-rank test. Operator 1, *z* = −1.07, *p*=0.29. Operator 2, *z* = −0.22, *p*=0.83).  Figure 2 shows the relationship between the change in Ko after crosslinking (i.e., preop value minus postop value) compared with the Ko estimated before crosslinking. Linear regression revealed significant associations between the change in Ko values following CXL (*ΔKo*) and the Ko revealed prior to CXL treatment (*x*).

(3) $$\begin{array}{c} \Delta Ko=0.945x-0.018\left(r=0.711,n=18,p<0.01\right), \end{array}$$

for the results obtained by operator 1, and

(4) $$\begin{array}{c} \Delta Ko=0.880x-0.016\left(r=0.935,n=20,p<0.01\right), \end{array}$$

for the results obtained by operator 2.

There was a significant correlation between the change in Ko following CXL (*ΔKo*) treatment and the number of days elapsed since treatment was carried out (*x*) according to the results from operator 1. This is best summarised as follows:

(5) $$\begin{array}{c} \Delta Ko=0.014-0.0002x\left(r=0.481,n=18,p=0.043\right). \end{array}$$

A similar significant correlation was not revealed for the results obtained by operator 2.

## 4. Discussion

The coefficient of corneal rigidity (Ko) in normal eyes ranges from 0.005 to 0.040 [9] and this is supported by most of the results shown in Table 1. The Ko values in Table 2 are comparable with other reports for keratoconus and disease-free normal corneas assessed using Friedenwald's technique [4–6, 11–15] and those obtained using methods based on ocular penetration [29–31]. Previous investigators tended to rely on parametric statistical tests when comparing Ko values based on Friedenwald's procedure [4–6, 11–15].  Table 1 clearly shows that on some occasions the Ko value was ≥0.11, way beyond the expected range. These outliers accounted for about 8% of the total number of measurements obtained, so it was decided to exclude these data from any further analysis. However, excluding these outliers still revealed that Ko values were not normally distributed, except for the values obtained by operator 1 from the control group. Overall, it was not appropriate to subject Ko data to parametric statistical tests. The results do not endorse the use of the parametric statistical tests instigated by earlier investigators. Furthermore, earlier investigators made no mention of encountering similar outliers.

Figure 1 shows the estimation of Ko was operator-dependent where the limit of agreement was ±0.039. This value, ±0.039, is approximately double the typical Ko value reported in Table 2. There are two clear outliers in Figure 1. Excluding these two from the analysis reduces the limit of agreement to ±0.021, nullifies the significance of the regression line, and the limit of agreement are now on par with most Ko values shown in Table 2. To the best of our knowledge, this is the first report of the interoperator limit of agreement associated with Friedenwald's technique.

A casual glance over the figures in Table 2 implies there might be significant interoperator differences in the estimation of Ko. But the differences were small, not statistically significant, and the procedure failed to distinguish between the three groups. The pachymetric data clearly shows the keratoconus cases were markedly different when compared with the controls. Yet, Friedenwald's technique did not differentiate between keratoconus and control cases. There were other discrepancies in the findings. For example, Ko tended to be lower in keratoconus and higher in the controls according to the results obtained by operator 1. This was expected, but the opposite was encountered for the results obtained by operator 2. Inconsistencies have also been encountered when Ko has been estimated by other means. For example, using an ocular penetrative technique in normal eyes, the mean (±sd) values for Ko ranged from 0.0126 (±0.012) to 0.0218 (±0.0053) [29–31].

Table 3 shows that intersessional differences in the estimation of mean Ko values were not significant. This implies that intersessional differences are small and inconsequential. The significance of equation 2 shows that, according to data obtained by operator 2, an intersessional shift in Ko could be small but it is predictable on a case-by-case basis. For the two operators, the RMS intersessional differences in Ko were 0.011 and 0.012. A measured change in Ko would need to exceed these values to be considered relevant.

CXL increases the rigidity of the cornea over the tangential plane but not along the sagittal depth [32–35]. Stromal lamellae retain the ability to slide against each other during corneal indention because interlamellar cohesive forces are not affected by CXL [35]. Some biomechanical properties of the cornea estimated by deliberately indenting the cornea should be either temporary or remain unchanged after CXL, and this has been confirmed in some studies [36–44]. It would be reasonable to expect Figure 2 to consist of an amorphous cluster of data points with no obvious correlation between ‘x' and ‘y' values if the facility for stromal interlamellar sliding remained unchanged after CXL. This was not the case. As shown in Figure 2, according to the results independently obtained by the operators, the change in Ko following CXL was highly dependent upon the Ko value prior to the CXL treatment. Furthermore, the concordance of the two best fit lines in Figure 2, and the similarity of indices in equations (3) and (4), imply that Ko is likely to increase after CXL when the preop value is < 0.018, and the opposite should occur when the preop value is > 0.018. The relatively flaccid cornea is likely to harden after CXL, but controversially, the relatively stiffer cornea is likely to soften. Yet, for those cases that did not receive any CXL treatment between sessions, equation 2 also predicts an increase in Ko when the value at session 1 is < 0.018 and a fall when this is > 0.018. According to results obtained by operator 2, on an individual case-by-case basis, the change in Ko following CXL treatment was on par with the change in Ko in cases that did not receive CXL between sessions. Thus, it cannot be concluded that the apparent changes in Ko following CXL were due solely to the treatment. The changes in Ko from time to time may have been due to other factors, or errors, in the estimation of Ko. However, according to the results obtained by operator 1, there was no correlation between the intersessional change in Ko in cases that did not receive any CXL treatment between measurements, but there was a weak though significant link between the change in Ko and the time elapsed since CXL treatment was meted out as described by equation (5). This was not supported by the results obtained by the other operator, yet another indication that Friedenwald's technique is operator-dependent. The RMS repeatability of Ko between sessions was 0.011 for operator 1 and 0.012 for operator 2. A difference in Ko estimated using Friedenwald's technique could be considered relevant if it exceeds these values.

## 5. Conclusion

Friedenwald's technique for estimating the coefficient of rigidity of the cornea is operator dependant, has poor repeatability, and cannot distinguish between normal, keratoconus, and post-CXL corneas.

## Figures and Tables

**Table 1:** Distribution of Ko estimations according to results taken by the two operators from keratoconus, post-CXL, and normal controls. The number of Ko values (proportion of total in parenthesis) per Ko range. K-S = Result of Kolmogorov–Smirnov test for normality of data in each column. ^*∗*^Indicates where the significance of the K-S statistic was <0.05 after excluding Ko values ≥ 0.11.

	Operator 1			Operator 2		
Ko range (mmHg/*μ*L)	**Keratoconus**	**Post-CXL**	**Normal**	**Keratoconus**	**Post-CXL**	**Normal**
0–0.01	9 (0.18)	7 (0.18)	1 (0.05)	6 (0.12)	9 (0.23)	1 (0.05)
0.011–0.02	21 (0.41)	15 (0.38)	9 (0.45)	24 (0.47)	20 (0.50)	10 (0.50)
0.021–0.03	14 (0.28)	11 (0.28)	2 (0.10)	11 (0.22)	5 (0.25)	5 (0.25)
0.031–0.04	5 (0.10)	4 (0.10)	4 (0.20)	2 (0.04)	1 (0.025)	1 (0.05)
0.041–0.05			1 (0.10)	2 (0.04)	2 (0.05)	1 (0.05)
0.051–0.06				1 (0.02)		
0.061–0.07						
0.071–0.08						
0.081–0.09				1 (0.02)		
0.091–0.10						
≥0.11	2 (0.04)	3 (0.08)	3 (0.15)	4 (0.08)	3 (0.075)	2 (0.10)
K-S	0.262^*∗*^	0.348^*∗*^	0.199	0.340^*∗*^	0.289^*∗*^	0.387^*∗*^

**Table 2:** Interoperator and intergroup differences in Ko estimations. 1 = Ko values estimated by operator 1. 2 = Ko values estimated by operator 2. Figures in parentheses are the number of individual cases where Ko values were not ≥0.11.

	Mean	Median	Mode	±sd	95% CI	Range
1 keratoconus (47)	0.019	0.018	0.025	0.008	0.017–0.021	0.009–0.035
1 post-CXL (37)	0.019	0.020	0.022	0.007	0.017–0.022	0.007–0.035
1 normal (17)	0.022	0.019	0.019	0.010	0.017–0.027	0.011–0.041
2 keratoconus (47)	0.021	0.017	0.021	0.014	0.024–0.032	0.007–0.088
2 post-CXL (37)	0.018	0.016	0.020	0.009	0.015–0.021	0.007–0.047
2 normal (17)	0.019	0.019	0.011	0.007	0.015–0.022	0.009–0.035

**Table 3:** Intersessional differences in Ko estimations. 1 = Ko values estimated by operator 1. 2 = Ko estimated by operator 2. Figures in parentheses are the number of individual cases in each group.

	Mean	Median	Mode	±sd	95% CI	Range
1, 1^st^ session (27)	0.020	0.020	0.025	0.011	0.016–0.024	0.008–0.051
1, 2^nd^ session (27)	0.016	0.015	0.011	0.008	0.013–0.019	0.008–0.047
2, 1^st^ session (16)	0.016	0.013	0.015	0.012	0.010–0.021	0.006–0.054
2, 2^nd^ session (16)	0.018	0.013	0.025	0.012	0.012–0.024	0.007–0.047

## Data Availability

The data supporting the findings of this study are available from the corresponding authors upon request.
